# Supplementary material for: Public investments in the development of GeneXpert molecular diagnostic technology
Source: PLoS One. 2021 Aug 31;16(8):e0256883. doi: 10.1371/journal.pone.0256883 (PMC8407584; doi:10.1371/journal.pone.0256883)
Supplement: S2 File — (DOCX) [file pone.0256883.s002.docx]

**S2 File**

**Table 1. Databases of federal and philanthropic funding and search terms used**

| **Database** | **URL** | **Search terms used** |
| --- | --- | --- |
| NIH RePORTER | <https://projectreporter.nih.gov/reporter.cfm> | “Cepheid”, “GeneXpert”, and “David Alland” (David Alland’s laboratory is known to have been closely involved with GeneXpert development) |
| Small Business Innovation Research (SBIR) and Small Business Technology Transfer (STTR) programs | <https://www.sbir.gov/sbirsearch/award/all> | “Cepheid”, “Alland”, “Northrup”, “Lawrence Livermore”, “GeneXpert” |
| U.S. Department of Defense Grant Awards Website. Available from: (only covers grants back to 2014 due to 2015 DoD Appropriations Act (Division C of the Consolidated and Further Continuing Appropriations Act, Public Law 113-235)) | <https://dodgrantawards.dtic.mil/grants> | “Cepheid”, “GeneXpert”, “Alland” |
| Department of Defense. Congressionally Directed Medical Research Programs. | <https://cdmrp.army.mil/search.aspx> | “Cepheid”, “GeneXpert”, “Alland” |
| beta.SAM.gov | <https://beta.sam.gov> | “Cepheid”, “GeneXpert” |
| USAspending: Government Spending Open Data | <https://www.usaspending.gov> | “Cepheid”, “GeneXpert” |
| Bill & Melinda Gates Foundation | Bill and Melinda Gates Foundation. Awarded Grants. Available from: <https://www.gatesfoundation.org/How-We-Work/Quick-Links/Grants-Database/> | “Cepheid”, “GeneXpert” |
| Biomedical Advanced Research and Development Authority (U.S. Department of Health & Human Services) | BARDA’s Rapidly Expanding  COVID-19 Medical Countermeasure Portfolio. <https://medicalcountermeasures.gov/app/barda/coronavirus/COVID19.aspx> | “Cepheid” |

**Table 2. Sources cited for public sector investments in GeneXpert.**

| **Year** | **Project name** | **Funder(s)** | **Amount (USD, inflation-adjusted to 2020)** | **Source** |
| --- | --- | --- | --- | --- |
| Pre-1996 | Development of underlying technologies at Lawrence Livermore National Laboratory before creation of Cepheid | U.S. Department of Energy, U.S. Department of Defense (DARPA, U.S. Army) | ≥10,674,919 | Northrup Consulting Group. Awards and Funding Sources. http://northrupconsultinggroup.com/awards.html (accessed Sept 27, 2020). |
| 1998-2006 | SBIR/STTR funding | Department of Health and Human Services, U.S. Department of Defense (U.S. Army) | 9,601,010 | NIH RePORTER and SBIR.gov |
| 1997-2020 | NIH funding of university research that contributed to GeneXpert development | U.S. Department of Health and Human Services (NIH) | 42,077,663 (pre-market development) +  13,732,770 (real-world validation studies) | NIH RePORTER: search terms "Cepheid", "GeneXpert", "David Alland", duplicates removed. |
| 1996-2020 | R&D tax credits | U.S. federal and state governments | 66,815,060 | Estimated based on information in financial statements, see relevant section in the Appendix. |
| Before 2001 | Micro-fluidic Integrated DNA Analysis System (MIDAS) | U.S. Department of Defense (Edgewood Research, Development and Engineering Center) | 3,455,708 | Page 160. North American Technology and Industrial Base Organization. February 2001. Biological Detection System Technologies: Technology and Industrial Base Study. Available from: https://apps.dtic.mil/dtic/tr/fulltext/u2/a438853.pdf |
| 1997 | US Army 'specified device' | U.S. Department of Defense (U.S. Army) | 8,141,691 | Cepheid. 10-K filing for fiscal year 2002. Available from:https://www.sec.gov/Archives/edgar/data/1037760/000113626103000007/body10k.htm |
| 1998 | DARPA 'specific device' | U.S. Department of Defense (DARPA) | 6,191,523 | Cepheid. 10-K filing for fiscal year 2002. Available from:https://www.sec.gov/Archives/edgar/data/1037760/000113626103000007/body10k.htm |
| 2000 | Soldier Biological Chemical Command project | U.S. Department of Defense (Soldier Biological Chemical Command) | 2,649,719 | Cepheid. 10-K filing for fiscal year 2002. Available from:https://www.sec.gov/Archives/edgar/data/1037760/000113626103000007/body10k.htm |
| 2003 | USPS BDS Program – Northrop Grumman consortium – first phase | U.S. Postal Service | 36,409,637–41,265,255 | Cepheid. 10-K filing for fiscal year 2014. Available from:<https://www.sec.gov/Archives/edgar/data/1037760/000119312515065981/d852874d10k.htm> ; <https://sst.semiconductor-digest.com/2004/04/postal-service-delays-rollout-of-cepheids-anthrax-detector/> ; Cepheid’s share estimated based on 15-17% of U.S. $175 million (estimate made in Cepheid. 10-K filing for fiscal year 2003. Available from:https://www.sec.gov/Archives/edgar/data/1037760/000104746904007707/a2129996z10-k.htm ), midpoint of 16% ($38,837,446) used for simplicity. |
| 2006 | Xpert MTB/RIF assay development | FIND | 8,936,677 | Cepheid. 10-K filing for fiscal year 2014. Available from:https://www.sec.gov/Archives/edgar/data/1037760/000119312515065981/d852874d10k.htm |
| 2006 | CDC influenza POC test | U.S. Department of Health and Human Services (CDC) | 3,857,962 | Cepheid Inc. 10-K filing for fiscal year 2006. Available from:<https://www.sec.gov/Archives/edgar/data/1037760/000095013407005786/f28285e10vk.htm> ; Cepheid Inc. 10-K filing for fiscal year 2007. Available from:https://www.sec.gov/Archives/edgar/data/1037760/000095013408003850/f38496e10vk.htm" |
| 2011 | Xpert HIV-1 VL assay development | FIND | 5,965,551 | Cepheid. 10-K filing for fiscal year 2014. Available from:https://www.sec.gov/Archives/edgar/data/1037760/000119312515065981/d852874d10k.htm |
| 2011 | GeneXpert remote calibration kit | FIND | 1,169,716 | Cepheid. 10-K filing for fiscal year 2012. Available from:https://www.sec.gov/Archives/edgar/data/1037760/000119312512063367/d272777d10k.htm |
| 2014 | Xpert MTB/RIF Ultra assay development | FIND | 3,314,496 | Cepheid Inc. 10-K filing for fiscal year 2014. Available from:https://www.sec.gov/Archives/edgar/data/1037760/000119312515065981/d852874d10k.htm |
| 2014 | Xpert Ebola assay development | Paul G. Allen Family Foundation and the Bill & Melinda Gates Foundation | 3,756,428 | Cepheid Inc. 10-K filing for fiscal year 2014. Available from:https://www.sec.gov/Archives/edgar/data/1037760/000119312515065981/d852874d10k.htm |
| 2016 | Finger-stick HIV viral load blood test | Bill & Melinda Gates Foundation | 4,645,887 | Bill and Melinda Gates Foundation. Awarded Grants. Available from: https://www.gatesfoundation.org/How-We-Work/Quick-Links/Grants-Database/ |
| 2017 | Xpert MTB/XDR assay development | FIND | 2,122,481 | TAG TB R&D Survey – Cepheid response in 2017. |
| 2017 | Finger-stick TB triage blood test | U.S. Department of Defense (U.S. Army) | 3,788,788 | U.S. Department of Defense Grant Awards Website. Available from: <https://dodgrantawards.dtic.mil/grants> |
| 2020 | Xpert SARS-CoV-2 assay development | U.S. Department of Health and Human Services (BARDA) | ≥4,700,000 | U.S. Department of Health & Human Services. COVID-19 Medical Countermeasure Portfolio. Available from: <https://medicalcountermeasures.gov/app/barda/coronavirus/COVID19.aspx> |
| 2007-2020 | FIND expenses on collaborative projects with Cepheid developing GeneXpert technology, not captured above, including the Omni platform | FIND | $6,740,785 for clinical and laboratory studies, $19,688,602 on other research and development costs, and $3,125,175 on relevant FIND internal operating expenses, such as staff and travel costs. | Personal communication, email from FIND to David Branigan, 14th of January 2021. |

**Tax credits**

Cepheid has reported net operating losses in all fiscal years (FY) since the company's inception in 1996—excepting meagre (~$0.5 million) net profits in 2011 and 2015—up to its acquisition by the multinational Danaher in 2016. As a result, Cepheid has had zero or very low income taxes to pay in all tax years 1996-2016.

In FY 2015 (last Cepheid filing year before the Danaher acquisition), Cepheid reported: "As of December 31, 2015, the Company had federal research and development tax credits of $13.1 million, which expire in the years 2018 through 2035, and state research and development tax credits of $15.2 million, which carry forward indefinitely. The Company also had foreign tax credits of $1.6 million which expire between 2020 through 2025.”

Danaher, which owns Cepheid since 2016, has made large profits every FY (billions), and has reported substantial income tax payments (hundreds of millions). It would therefore be reasonable to assume that Danaher has been able to, or will be able to, take advantage of the full amount of the Cepheid credits reported as having accrued in/by FY 2015 ($29.9 million or $32.4 million inflation-adjusted to 2020 USD) because Danaher, unlike Cepheid, actually has income taxes to pay.

Tax credits pertaining to Cepheid R&D expenditures have not been reported in Danaher’s filings, meaning that data are not available on the five-year period 2016-2020. In order to fill this gap, we projected expected tax credits based on previous tax credits, as outlined below.

2015 is the last year for which reported R&D expenditures are available.^[[1]](#footnote-1)^ In order to estimate tax credits for 2015-2020, we first estimated Cepheid R&D expenditures for 2015-2020 by assuming that they continued increasing in a linear trend based on expenditures in 2010-15. This yields total estimated R&D expenditures over 2016-2020^[[2]](#footnote-2)^ of $798,539,000 or $826,581,162 after inflation adjustment.

*Inflation-adjusted reported and projected annual R&D expenditures by Cepheid*

We then assumed that tax credits are proportional to R&D expenditures. In 2015 (last year reported), Cepheid reported tax credits of $29.9 million, which is equivalent to 4.16% of their total reported R&D expenditures over 1996-2015 ($29,900,000 / $718,579,000).

Applying this proportion—4.16%—to projected R&D expenditures over 2016-2020 (0.0416 x $826,581,162), we arrive at an estimate of $34,393,959 in tax credits gained over the 2016-2020 period.

Combining the reported and estimated tax credits ($32,421,101 + $34,393,959) gives a total tax credit estimate of $66,815,060 since creation of the company in 1996.

As the GeneXpert diagnostic platforms and compatible assays and software are Cepheid’s only products there is no need to calculate what proportion of tax credits are attributable to GeneXpert; it can be assumed to be 100%.

**Table 3. Government sponsored research funding reported in Cepheid annual reports (USD).**

| **Year** | **Government sponsored research** | **Contract revenues*** | **R&D expenditures** | **Total revenues** |
| --- | --- | --- | --- | --- |
| 1996/7 | 1,400,000 | 45,000 | 2,220,000 | 1,445,000 |
| 1998 | 2,870,000 | 707,000 | 5,990,000 | 3,577,000 |
| 1999 | 2,249,000 | 1,187,000 | 10,089,000 | 3,595,000 |
| 2000 | 2,249,000 | 416,000 | 14,810,000 | 7,062,000 |
| 2001 | 2,554,000 | 131,000 | 14,620,000 | 11,354,000 |
| 2002 | 1,838,000 | 403,000 | 16,356,000 | 14,654,000 |
| 2003 | 2,079,000 | 638,000 | 15,330,000 | 18,534,000 |
| 2004 | 34,000 | 2,967,000 | 15,903,000 | 52,968,000 |
| 2005 | 1,508,000 | 3,062,000 | 18,961,000 | 85,010,000 |
| 2006 | 1,036,000 | 3,913,000 | 23,886,000 | 87,352,000 |
| 2007 | 4,387,000 | 8,554,000 | 31,449,000 | 129,473,000 |
| **Total** | 22,204,000 | 22,023,000 | 169,614,000 | 415,024,000 |

Source: Cepheid 10-K filings, available on the EDGAR database, at https://www.sec.gov/edgar.shtml

‘Government sponsored research’ and ‘contract revenues’ were not reported in filings after 2007.

*’Contract revenues’ is a category of income reported in Cepheid’s annual filings, which appears to cover a wide range of income, including government grants and contracts. For example: *"Contract revenues. Contract revenues consist of fees earned under technology license arrangements, services rendered under research and development arrangements, grants and government sponsored research agreements, and milestone payments and royalties received under license and collaboration agreements. Deferred revenue is recorded when funds are received in advance of technologies to be delivered or services to be performed." https://www.sec.gov/Archives/edgar/data/1037760/000119312509038982/d10k.htm*

**Table 4. Public investments by disease category.**

| **Disease category** | **NIH funding** | **Other US government funding** | **Philanthropic funding*** |
| --- | --- | --- | --- |
| TB | $36,556,639 | $3,788,788 | $14,373,654 |
| bacterial disease (sepsis and biothreat agents) | $19,501,353 | – | – |
| COVID-19 | – | $4,700,000 | – |
| HIV | – | – | $4,645,887 |
| Ebola | $239,267 | – | $3,756,428 |
| Influenza | $3,857,962 | – | – |
| HPV | $3,727,623 | – | – |
| Cancer | $2,670,831 | – | – |
| Chlamydia | $1,442,302 | – | – |
| Cannot allocate to a disease category | – | $146,367,076** | $8,045,641 |

*Includes investments by FIND.

**Includes small contribution from NIH via SBIR-STTR.

1. https://www.sec.gov/Archives/edgar/data/1037760/000162828016011718/cphd12312015-10xk.htm [↑](#footnote-ref-1)
2. 5 years from start of 2016 to end of 2020. [↑](#footnote-ref-2)
