# Supplementary material for: Public investments in the development of GeneXpert molecular diagnostic technology
Source: PLoS One. 2021 Aug 31;16(8):e0256883. doi: 10.1371/journal.pone.0256883 (PMC8407584; doi:10.1371/journal.pone.0256883)
Supplement: Attachment — Submitted filename: TAG GeneXpert response to review 2021-06-11.docx [file pone.0256883.s004.docx]

Dear Editor and reviewers,

Thank you for your careful review of our submission and thoughtful comments, which have improved the manuscript. Please see below our point-by-point response to your comments. Please note line numbers refer to those in the marked-up document, not the ‘clean’ document.

We have also made the requested changes to formatting.

With sincere thank again,

Dzintars Gotham

**

**EDITORS’ COMMENT:**

Thank you for stating the following in the Competing Interests section:

DG received a research grant from TAG as an independent researcher, and DB, LM, and SM are employees of TAG. SD is an employee of Médecins Sans Frontières (MSF). TAG and MSF have long advocated for lowering the price of GeneXpert tests in low- and middle-income countries. The authors declare no other competing interests.

We note that you received funding from a commercial source: Treatment Action Group

Please provide an amended Competing Interests Statement that explicitly states this commercial funder, along with any other relevant declarations relating to employment, consultancy, patents, products in development, marketed products, etc.

Within this Competing Interests Statement, please confirm that this does not alter your adherence to all PLOS ONE policies on sharing data and materials by including the following statement: "This does not alter our adherence to PLOS ONE policies on sharing data and materials.” (as detailed online in our guide for authors http://journals.plos.org/plosone/s/competing-interests). If there are restrictions on sharing of data and/or materials, please state these. Please note that we cannot proceed with consideration of your article until this information has been declared.

Please include your amended Competing Interests Statement within your cover letter. We will change the online submission form on your behalf.

**AUTHORS’ RESPONSE:**

If we have understood correctly, the only request here is to spell out TAG as ‘Treatment Action Group’. We have done so in the revised manuscript (lines 899-904).

**REVIEWER #1 COMMENT:**

Reviewer #1: This study looks at the public and philanthropic/not-for profit contributions to the development of the GeneXpert technology. This is an important compliment to the work that has already been done examining the public contribution to the development of pharmaceuticals. Having made their case that there was a substantial amount of public money invested I would like to read some policy recommendations about going forward, e.g., tying public investment to pricing commitments.

**AUTHORS’ RESPONSE:**

Thank you. We have added some policy recommendations into the Discussion in a new sub-heading, lines 512-559.

**REVIEWER #1 COMMENT:**

1. The authors make the case about the value of the GeneXpert test for TB and should provide some metrics to back up their statement e.g.., false positive and false negative rates and how do they compare with the metrics from the older more traditional tests? Similarly, how do the costs for the GeneXpert test compare to the costs for the older tests both in high-income countries like the US and in low- and middle-income countries?

**AUTHORS’ RESPONSE:**

The key metric that differentiates GeneXpert TB tests from earlier sputum smear microscopy tests is sensitivity. We have included the sputum smear microscopy test sensitivity of 65% in comparison to the GeneXpert TB test sensitivity of 90%, with the appropriate referencing of WHO-coordinated systematic reviews of diagnostic accuracy studies. The key metric that differentiates GeneXpert TB tests from culture is time to results, which is already stated. (lines 60-71)

We have included reference to the cost of GeneXpert TB tests in eligible LMICs at $9.98 compared to the average cost of sputum smear microscopy at $3.83, to highlight the difference in cost to country programs, which has contributed to limited scale up of rapid molecular testing to replace sputum spear microscopy in high-TB-burden countries. (lines 504-508).

**REVIEWER #1 COMMENT:**

2. Did the WHO convene a committee to evaluate (and endorse) the test? If there was a committee did the membership have to declare their conflicts and is that information available?

**AUTHORS’ RESPONSE:**

Yes, the WHO did convene independent Expert Groups that declared conflicts of interest for each of the WHO policy endorsements of GeneXpert TB tests. These declarations of interests are reported and publicly available in the corresponding WHO policy documents. We have included a sentence that states this. (lines 79-80)

**REVIEWER #1 COMMENT:**

3. Line 120: Explain what the Treatment Action Group is.

**AUTHORS’ RESPONSE:**

Thank you, we have added a note on line 143.

**REVIEWER #1 COMMENT:**

4. On page 11 the authors mention data from SEC filings, but this is not described in the Methods. In addition, did the authors search the financial press, e.g., Financial Times, Wall Street Journal?

**AUTHORS’ RESPONSE:**

In the Methods, we note that “Cepheid’s annual financial reports (1996-2016) were reviewed”. The SEC references on page 11 refer to annual (10-K) reports filed with SEC. We have now aligned wording in these two sections to make this clearer.

(See lines 138-139 in Methods and lines 276-277 in Results).

We did not review the financial press.

**REVIEWER #1 COMMENT:**

5. Line 139: What do the authors mean by "relevant materials"?

**AUTHORS’ RESPONSE:**

This refers to materials published by Cepheid. We have rearranged the sentence to make this clear (lines 170-173).

**REVIEWER #1 COMMENT:**

6. On lines 223-224, the authors spell out DARPA in full, but that should be done the first time DARPA is mentioned on line 171.

**AUTHORS’ RESPONSE:**

Thank you, this is now corrected (line 210).

**REVIEWER #2 COMMENT:**

Reviewer #2: It was a pleasure to read this thoroughly research and convincingly argued article. Drawing upon multiple sources of financial data and other documentation to elucidate the development of GeneXpert -- and the vast public resources that went into it --, the authors explain in meticulous detail the complex pathway, funding structures, and array of actors, from which this critically important public health intervention has emerged. The article also represents a novel contribution to the literature in that, although similar studies have been done on drugs and biologics (e.g., https://doi.org/10.1093/jlb/lsz019), to my knowledge no such study has been undertaken with respect to a medical device. I also found the authors' incorporation of key informant feedback into their analysis, to corroborate and triangulate what the documents suggest, to underscore the depth of their approach and importance of their findings. In short, Bravo!

There were, however, a few minor points, which I wanted to highlight for the authors' consideration. Space may pose a challenge to incorporating them all. But to the extent they can do so, I think it will further enhance an already strong article.

i) Can the authors clarify (in the body of the manuscript) how the tax credits (p. 13, line 267) were calculated? Tax credits are often overlooked in this kind of analysis; so that others might follow suit in the future, it would be helpful to know exactly how the authors arrived at this figure.

**AUTHORS’ RESPONSE:**

Thank you – we outline the calculation of tax credits in the Appendix. We have now added a note in Methods and in Table 1 to direct the reader to the Appendix for details on R&D tax credit estimation (lines 296).

Also, we have added a citation for the paper on Ebola vaccine R&D investments (indeed a very important case!) on line 118.

**REVIEWER #2 COMMENT:**

ii) I thought there was room to expand the critique in the discussion. The authors' attention to the buy-down agreements is key, especially given that will expire in 2022. However, such agreements seem like a band-aid solution unable to address the fundamental problem that the public's investments in GenXpert were in no way reflected in the many funding agreements, contracts, etc. that attended and structured its development. I would encourage the authors to make this point explicit in some way, space permitting.

**AUTHORS’ RESPONSE:**

Thank you for this comment. We agree that the public’s investments were not reflected in the many funding agreements between public/nonprofit funders and Cepheid, except for the 2006 FIND-Cepheid agreement, which included provision for COGS+ pricing and volume-based price adjustments. We’ve included clear position statements on the need for accountability to the public for fair pricing of health products developed through public investments. We’ve also highlighted the insufficiency of the buy-down as a short-term band-aid solution that did not put in place conditions to promote long-term access. (Lines 471-473 and lines 516-525)

**REVIEWER #2 COMMENT:**

iii) Similar to point ii), I think it would be useful (again, space permitting) to allude to potential policy responses to the problem. There is a fairly extensive literature regarding the lack of use of "march-in" rights related to NIH funding. Can the authors point to that literature and/or develop an argument as to why such rights could or will not work in this case? Another mechanism that has received attention is section 1498 of the Patent Act, which would allow the US govt to override patent rights (see e.g., https://papers.ssrn.com/sol3/papers.cfm?abstract_id=3685413). Patents seem to be in place here. Does 1498 provide a potential solution? Like march-in rights it has seldom been used in recent years. Perhaps that highlights another layer of the problem; namely, that the state, through agencies like NIH, is at ease with the status quo and will not push back against the dominant logics of commercialization. Again, I raise this point only to underscore the importance of the article's findings -- not as a critique. By highlighting the non-use of policy tools at the government's disposal, it may raise additional questions to pursue in the future.

**AUTHORS’ RESPONSE:**

We have added a point on compulsory licensing and march-in rights, in lines 539-543.

**REVIEWER #2 COMMENT:**

iv) Finally, relating to patents, I am a little unclear about the patent landscape that the authors perform. Can the authors clarify that aspect of their research a little? I think that would be helpful, especially if details regarding policy tools that pertain to patents, are integrated in a revised version of the article.

**AUTHORS’ RESPONSE:**

We did not undertake a patent landscape analysis, but refer to an existing patent landscape that was commissioned by the civil society organisation Public Citizen (reference 20 in the manuscript, line 178):

Public Citizen. GeneXpert Patent Landscape. 2014. https://www.citizen.org/article/genexpert-patent-landscape/ (accessed 20 Jan 2021).

**REVIEWER #3 COMMENT:**

Reviewer #3: I would like to command the authors for this innovative research idea and execution of the work. The authors’ work provides a comprehensive historical overview and estimated grand total of public investments in the development of the GeneXpert technology and Xpert cartridges. These information are very useful in understanding the role in public investments in facilitating the innovation in technologies (diagnostic) for infectious disease, particularly in those disease (e.g. Tuberculosis) that lack considerable private and overall research and development investments.

This reviewer finds this article can benefit from following revisions in presentation of the authors research methodology and findings.

1) It will be important for authors to provide specifics of data identification and extraction process:

**AUTHORS’ RESPONSE:**

The Appendix (S2 file) provides links to databases searched and keywords used. We searched all databases of grants by (relevant) U.S. federal agencies that we could identify. There is no comprehensive list of US grant agencies that we are aware of.

**REVIEWER #3 COMMENT:**

a. What specific criteria was use to include/exclude information used for this work

**AUTHORS’ RESPONSE:**

As mentioned in response to a different point above, this element was made easier by the fact that the GeneXpert platform is the only product commercialised by Cepheid, so all grants to Cepheid for research/technology development were considered relevant to GeneXpert.

For research grant databases, we included grants that explicitly mentioned GeneXpert or Cepheid by name, as well as grants given to a key researcher (David Alland), whose lab is known to have collaborated extensively with Cepheid in developing the technology, where the grant abstract clearly described work on a point-of-care PCR-based diagnostic (or mentioned Cepheid or GeneXpert). We have added this clarification in lines 157-160.

Grants were included as relevant to GeneXpert development where the project focused on developing/improving the GeneXpert device or assay itself (rather than used the GeneXpert test to address a different research question). Grants were classed as ‘validation’ studies where the grant focused on evaluating the performance of an assay in a real-world setting (we have added sentences to this effect in Methods, lines 160-165).

The full data are available in the Appendix (S1 file): NIH RePORTER and other database data extracts including abstracts, and our coding for whether or not each grant pertained to GeneXpert development/validation.

We have added information on the number of grants identified and number coded as relevant, in lines 300-303.

**REVIEWER #3 COMMENT:**

b. Whether there were ‘duplicates’ of information retrieved from multiple databases and reports and how these were screened/identified, and included as final estimates reported in this study?

**AUTHORS’ RESPONSE:**

By design, we would not expect overlap (i.e. duplicates) between the databases as they are agency-specific. One exception is SBIR-STTR grants, for which there was overlap between NIH and SBIR-STTR databases (see S1 Appendix); duplicate values were censored (excluded from summation) in order to avoid double-counting.

For the NIH search results, results from various search terms were de-duplicated based on project number (see also response to question above).

Cepheid reports ‘government R&D grants’ in their 10-K reports – and this would presumably overlap with many of the other identified grants. However, we ultimately ignored Cepheid’s reported government R&D grants because we considered our project-by-project survey to be better defined and more comprehensive. We have added text to explain this point on lines 281-293 and copied this new text below for the Reviewer’s convenience:

*“For the period 1996-2007, Cepheid reported annual summary values for ‘government sponsored research‘ and ‘contract revenues’ (the latter of which, by the company’s definition, includes research grants, among other things; see Table 3 in S2 Appendix). Over this period, Cepheid’s reports a total of $22,204,000 for government sponsored research and $22,023,000 for contract revenues. Direct comparison of these values to the totals identified by our survey (Table 1) is limited by the fact that many of the investments identified in our survey funded work not only by Cepheid, but by collaborators such as the University of Medicine and Dentistry of New Jersey. Additionally, it is not clear what proportion of ‘contract revenues’ are from research grants, and the company has not reported on these items after 2007. Due to these limitations and to avoid double-counting with our project-by-project survey (Table 1), we do not include these poorly-defined summary figures in our totals of public investment, or in our overview in Table 1 and Figure 1.”*

**REVIEWER #3 COMMENT:**

c. An overview of types of data/information extracted across all data source (the authors provide some of this information in the text, but it’s very difficult to assess this across multiple databases/sources) – the main idea for this is to show what type of information/data is available from each data source/database and this can help advocate the need for transparency and synchronization of financial information for publicly funded R&D activities

**AUTHORS’ RESPONSE:**

Thank you. Definitely this sort of overview makes sense; in fact an earlier draft of the manuscript did have such a Table which we edited out before submission. We have reinserted (and updated) this Table now – on lines 591-597.

**REVIEWER #3 COMMENT:**

d. If multiple screeners/data extractors were used for the study, how were the discrepancies of data interpretation resolved?

**AUTHORS’ RESPONSE:**

As the volume of records to be screened was relatively low and considered unambiguous, we did not use multiple screeners.

**REVIEWER #3 COMMENT:**

e. For certain data sources, it is possible that total funding/investment amount may be for multiple purposes (i.e. not exclusive to development of the GeneXpert systems/cartridges). Did any of the estimates which the authors included in the manuscript fall in to such criteria? If so, how were the cost/funding estimates apportioned to GeneXpert system/Xpert cartridge development (or was it that because fundings were specific to Cepheid, authors assumed that the full funding amount was for GeneXpert/Xpert cartridge)? If latter, it would be important to specify this decision in the methods section.

**AUTHORS’ RESPONSE:**

Thank you, this is a key point to clarify. Cepheid does not have any products apart from GeneXpert (see <https://www.cepheid.com/>). We therefore assumed 100% of public sector funding to be pertinent to GeneXpert development. If they did have other products, this could pose a methodological challenge for allocating funding between products, but that is not the case. We have added text to make this point explicit on lines 132-135.

**REVIEWER #3 COMMENT:**

2) Following up on the comment made in part c of #1 above, inclusion of an illustration/figure summary or a table to provide an organized overview of all sources of information (e.g. databases, financial reports) would help the readers in understanding the sources of data, types of data available from each source, and which of these public funding are relevant to each of of the main phases of the research and development processes of the GeneXpert and Xpert cartridge technologies [the authors break this process as three categories, but this reviewer would like to suggest four distinct categories: 1. development of modern PCR techniques; 2. miniaturization and automation of PCR machinery (specially for GeneXpert system), development of the cartridges (by cartridge purpose and disease type), and clinical research studies for field applications (this also specify by disease type)]

**AUTHORS’ RESPONSE:**

Thank you for this suggestion: We have added a new Table (Table 2, line 591) to outline the various information sources, data available from each, and data gaps (the latter ties in nicely with later discussion on lack of transparency etc.).

With regarding to breaking down funding by research phase, please see response to the next comment, directly below.

**REVIEWER #3 COMMENT:**

3) Table 1: The authors have constructed this table with an historical overview in mind and it’s very clear way to present how much investments were made at what time period, by whom. However, this reviewer feels that it would be much more useful to present this information by R&D process (including clinical trials), by product type (GeneXpert system vs. Xpert cartridges), and by purpose of the product’s use (e.g. by disease type). In other words, it will be very helpful to know that of 252 million dollar total public investment, what was the distribution of investment into specific R&D activities for each Cepheid GeneXpert ‘product’ that is currently in market?

**AUTHORS’ RESPONSE:**

Thank you for this suggestion. We have done our best to implement this suggestion, but are only partially able to provide the breakdowns suggested by the reviewer.

We have added an outlining breakdown by diseases focus on lines 380-386 (although the majority of funding, such as Cepheid’s R&D tax credits, cannot be broken down by disease focus).

For most of the funding identified, we do not have information on what proportion was spent on clinical trials, pre-clinical engineering work, work relating to securing regulatory approval, etc.. The exception is NIH grants, where what we have labelled as grants for ‘Validation’ work essentially represents clinical trials. (The concept of ‘clinical trials’ in diagnostic development normally takes the form of a cohort study in which the new diagnostic is compared to an existing gold standard.) For investments by FIND, we report in the Results section that across FIND’s collaboration with Cepheid, “FIND expenses on these projects totaled $6,740,785 for clinical and laboratory studies, $19,688,602 on other research and development costs, and $3,125,175 on relevant FIND internal operating expenses, such as staff and travel costs.” (FIND did not break down these figures per project.) Separation by R&D phase is not possible based on available data for the other philanthropic funding, or other US agency funding.

With regard to separating investments in development of the device versus assays: Based on the funding we identified in the analysis, it appears that the from around 2006 all or nearly all public funding has been for assay development with the exception of R&D tax credits, which are effectively equivalent to a proportion of the total R&D expenditures over the lifetime of the company (i.e., contribute to both device and assay development). We re-reviewed all NIH grant abstracts captured in our search. Nearly all NIH grants (based on analysis of grant abstracts) have been for assay development. Only one (4-year, 8.5 million USD from NIH) grant was clearly contributing to device development as well as assay development. (A new column has been added into the supplementary data spreadsheet to indicate our coding of whether a grant pertains to work on the device, assay, or both.) We have added a note on this in lines 369-371.

We have also added a note into the Limitations section on how investments could not be fully allocated to R&D stage / disease focus (lines 671-674).

**REVIEWER #3 COMMENT:**

Following are additional suggestions which the authors can revise/improve their discussion to contextualize the authors’ work in the current debate of transparency in R&D investments and public pricing of GeneXpert systems and cartridges in the high burden, lower income settings.

1) Can the authors comment (or perform additional analyses to estimate) how readers should view/interpret the $252 million dollars in public investment can further justify reduction (or at least continuing the 2012 buy down agreement? The authors provide detailed assessment of current buy down agreement, advocate’s perspectives in further reducing the price (or at least continuing the current agreement), but this reviewer feels it’d be important to find a way to link the authors’ work into this issue.

**AUTHORS’ RESPONSE:**

Thank you, we have added our position on this question into the Discussion in lines 612-659, arguing that GeneXpert technology should be shared with other manufacturers, and Cepheid should adopt a COGS-plus and volume based pricing structure.

**REVIEWER #3 COMMENT:**

2) What do the authors think about following issue? The current buy down agreement includes at least 20% profit margin for each unit/cartridge sold/procured (and this does not count potentially larger profit gains for units sold in countries outside of the buy down agreement), which roughly marks 100 million ($2 profit per Xpert MTB/RIF cartridges sold x 50 million so far procured globally) in return on their R&D investment (this does not include any other profit margins made in other global sales – GeneXpert systems, other cartridge types, and sales in non-buy-down agreement countries or in private sectors). How should readers view the public return on investment for the $252 million on R&D of GeneXpert and Xpert cartridges for infectious disease? It would be very interesting to provide some perspectives as to how this public investment in the R&D of GeneXpert should be represented in public pricings of GeneXpert related products, especially in countries with limited financial resources (i.e. simply put, if we were to estimate unit cost estimate for public investment as part of the cost of GeneXpert system and/or Xpert cartridges, what would this unit cost estimate for each cartridge procured in the span of 20 years (contextualized for typical duration of US patents)?)

**AUTHORS’ RESPONSE:**

Thank you very much for this comment. Indeed, it is important to be clear about how readers should view the public return on public investment in GeneXpert technology. Quantifying return on investment as a dollar figure per test for both the company and the public is a complex exercise with too many unknown variables. For diagnostics, the best public return on investment is fair pricing, which is COGS plus a reasonable/rational profit markup with volume-based price adjustments as economies of scale and manufacturing efficiencies are reached. Rather than quantifying a limited dollar figure, we rather prescribe fair pricing as the best public return on investment that promotes long-term sustainable access. In addition to the above-mentioned discussion of fair pricing added to the manuscript, we included a paragraph clearly stating how public return on public investment should be viewed. (Lines 533-542).

**REVIEWER #3 COMMENT:**

3) The authors discuss ‘limited transparency in R&D investments in the discussion section, but do not discuss how this transparency can be improved. This reviewer feels that the author’s work can provide some guidance (based on their experience in identifying and extracting data for this study) as to how this transparency issues can be improved, particularly for the public investment.

**AUTHORS’ RESPONSE:**

Thank you for this suggestion – we have added such recommendations in the ‘transparency’ section of the Discussion, lines 599-610.
